# No effects of acute tryptophan depletion on anxiety or mood in weight-recovered female patients with anorexia nervosa

**DOI:** 10.1007/s00406-022-01414-8

**Published:** 2022-05-05

**Authors:** Tomas Weinert, Fabio Bernardoni, Joseph King, Julius Steding, Ilka Boehm, Merle Mannigel, Franziska Ritschel, Florian Zepf, Veit Roessner, Stefan Ehrlich

**Affiliations:** 1 Translational Developmental Neuroscience Section, Division of Psychological and Social Medicine and Developmental Neuroscience, Faculty of Medicine, Technische Universität Dresden, Fetscherstraße 74, 01307 Dresden, Germany; 2 Centre and Discipline of Child and Adolescent Psychiatry, Psychosomatics and Psychotherapy, Faculty of Health and Medical Sciences, The University of Western Australia, Perth, WA Australia; 3 Telethon Kids Institute, Perth, WA Australia; 4 Department of Child and Adolescent Psychiatry, Psychosomatic Medicine and Psychotherapy, Jena University Hospital, Friedrich Schiller University, Jena, Germany; 5 Eating Disorder Research and Treatment Center, Department of Child and Adolescent Psychiatry, Faculty of Medicine, Technische Universität Dresden, Dresden, Germany

**Keywords:** Anorexia nervosa, Eating disorders, Anxiety, Tryptophan, Serotonin

## Abstract

**Background:**

Previous studies have suggested that individuals recovered from anorexia nervosa (AN) are characterized by increased serotonergic (5-HT) activity that might be related to elevated levels of anxiety. Assuming these traits to be also present in individuals at risk for AN, it was further hypothesized that restricting food intake might be a means to temporarily alleviate dysphoric affective states by reducing central nervous availability of tryptophan (TRP), the sole precursor of 5-HT. One study that supported this hypothesis found anxiolytic effects in individuals with a history of AN during an experimentally induced short-term depletion of TRP supply to the brain.

**Methods:**

In this placebo-controlled, double-blind cross-over study, 22 patients weight-recovered from AN (recAN) and 25 healthy control participants (HC) completed questionnaires assessing anxiety and momentary mood during acute tryptophan depletion (ATD), a dietary intervention that lowers central 5-HT synthesis.

**Results:**

The ATD procedure effectively reduced the ratio of TRP to competing for large neutral amino acids in the peripheral blood, indicating decreased TRP supply to the brain. Effects of ATD on anxiety and mood did not differ between recAN and HC. Bayesian null hypothesis testing confirmed these initial results.

**Discussion:**

Our results do not support the hypothesis that short-term depletion of TRP and its impact on the brain 5-HT reduces anxiety or improves mood in AN. As the evidence for the role of 5-HT dysfunction on affective processes in patients with AN is limited, further studies are needed to assess its relevance in the pathophysiology of AN.

**Supplementary Information:**

The online version contains supplementary material available at 10.1007/s00406-022-01414-8.

## Introduction

Anorexia nervosa (AN) is a serious eating disorder with prevalence estimates ranging between less than 1% and 4% among women, with a male:female ratio of approximately 1:10 [1]. It predominantly develops during adolescence with a peak incidence between 14 and 17 years of age [2], and is characterized by an intense fixation with body weight and self-starvation. Clinically, people affected by AN tend to be anxious, obsessional, perfectionistic, and harm avoidant even after recovery [3], indicating that these characteristics are not mere correlates of the acute illness but possibly independent premorbid traits [4, 5]. Despite ongoing research efforts, a specific pharmacological intervention that effectively reduces the mortality rate of AN (the highest for psychiatric disorders [6] and approximately 6 times higher than in the general population [7]) and alleviates unfavorable medical consequences [8] is not available. This may be due to a lack of understanding of the etiology of AN. For several years, the central nervous serotonin (5-HT) system has been proposed as a promising candidate to shed some light on the neurobiological basis of AN, and that it could be a valuable entry point for the development of more effective pharmacological interventions [3].

5-HT plays an important role in appetite regulation [3], and abnormalities in the 5-HT system (such as altered 5-HT availability) were found to be statistically associated with several traits and symptoms often found in individuals with or at risk for AN [3], including increased anxiety and low mood [9]. Indeed, several findings suggest alterations within the central nervous 5-HT system in acutely underweight and former AN patients: acutely ill patients show reduced tryptophan (TRP) plasma concentrations [10–12] and reduced concentrations of the 5-HT metabolite 5-hydroxyindoleacetic acid (5-HIAA) in peripheral blood plasma and cerebrospinal fluid (CSF) [13, 14]; individuals recovered from AN (recAN) were shown to have increased CSF 5-HIAA concentrations [15], increased platelet 5-HT content [16], and decreased monoamine oxidase (MAO-B) activity [17]. Furthermore, PET studies found altered 5-HT_1A_ and 5-HT_2A_ receptor bindings in recAN compatible with increased 5-HT activity and increased extracellular 5-HT concentrations [4, 18, 19]. Such changes in central 5-HT transmission seem to be associated with anxiety in AN [20], as was also confirmed by later studies [18]. Therefore, it has been hypothesized that patients with AN might seek temporary relief from dysphoric mood by reducing the availability of the 5-HT precursor and essential amino acid TRP by dieting [21]. In the following, we will refer to this notion as the ‘5-HT hypothesis of AN’. While suggesting a very basic mechanism that could motivate patients with AN to starve themselves, this hypothesis is also of interest for interventions in the neuropharmacological spectrum [3]. Assuming that persisting alterations of the central nervous 5-HT system in recAN reflect an underlying trait, a possibly fruitful research approach is to study such former patients to learn something about individuals at risk for AN.

As has been suggested for other neurotransmitters like dopamine [9], an inverted u-shaped curve might be a model that reflects associations between the brain 5-HT system and affective states, with either very high or low 5-HT availability leading to negative affective states. Based on this model, a reduction of central nervous TRP availability is expected to have a different effect on affect in recAN and possibly individuals at risk of AN compared to healthy individuals. Specifically, while a moderate reduction of TRP could restore optimal levels of 5-HT in individuals at risk for AN and improve anxiety and mood, it might reduce them to suboptimal levels in healthy controls (HC).

Acute tryptophan depletion (ATD) is a common experimental method that temporarily suppresses the supply of TRP across the blood–brain barrier by ingestion of a defined ratio of a TRP-free amino acid (AA) beverage containing large neutral amino acids (LNAAs) [22]. The reduction of TRP influx into the brain has been demonstrated to lead to decreased central 5-HT synthesis [23] and may therefore mimic the hypothesized effects of reduced food intake on the brain 5-HT system. Using this method, Kaye et al. [21] found that ATD had anxiolytic effects in young females with a history of AN but not in HC, thus providing support for the 5-HT hypothesis.

Despite the high relevance of the 5-HT hypothesis for the understanding of the neurobiological mechanisms of AN and the potential development of pharmacological interventions, the main original study [21] remains the only one in the field of AN using ATD and no known studies have reproduced the main findings. The present placebo-controlled double-blind cross-over study aims to fill this gap by assessing self-reported anxiety and mood changes following ATD versus placebo in recAN and HC participants. If the 5-HT hypothesis of AN is correct, we would expect that ATD has differential effects on self-reported affective states in former patients vs. HC individuals, i.e., decreased anxiety and less negative affect in recAN participants but not in HC.

## Methods

### Participants

A power analysis using G*Power [24] based on i) the within-group effect size found by Kaye et al. [21] (Cohens *d* = 0.90), ii) a Bonferroni-corrected α = 0.0125, and iii) a discovery power of 80% indicated a sample of 17 individuals in each group would be required to detect a significant within-group difference.

To ensure sufficient power even in the case of attrition, 49 female individuals were recruited for the present study: 22 recAN (17—26 years old) and 27 age-matched HC (17—26 years old). As determined with the SIAB-EX interview (see below), 20 recAN participants had been diagnosed with the restrictive subtype of AN during illness, two with binge/purge subtype. Two HC individuals had to be excluded: one due to scheduling difficulties and one due to subjective intolerance of the ATD procedure.

RecAN participants were recruited from our ongoing longitudinal study of AN and through advertisements in high schools and on the university campus. To be considered “recovered”, recANs had to 1) maintain a body mass index (BMI, kg/m^2^) > 18.5 (for participants of 18 years and older) or above the 10^th^ BMI-percentile with respect to their age (if younger than 18 years; Kromeyer-Hauschild et al., 2001) for at least 12 months prior to the study, 2) menstruate, and 3) refrain from binging, purging or other substantially restrictive eating patterns.

The control group consisted of normal-weight (BMI = 18.5–24.9 kg/m^2^ for adults and BMI above 10th percentile when under 18), eumenorrhoeic women. They were recruited via contact lists that were provided by the local municipality and advertising among high school and university students. We excluded HC if they reported any history of psychiatric illness as ascertained by a semi-structured psychiatric interview (see below).

We excluded participants if they had a lifetime history of any of the following clinical diagnoses: bulimia nervosa or binge eating disorder, schizophrenia, substance dependence, bipolar disorder, and suicidality. Further exclusion criteria for all participants were incomplete ATD/placebo beverage intake, nausea or other related intolerance of the ATD mixture, IQ lower than 85, organic brain syndrome, chronic medical or neurological illnesses that could affect appetite, eating behavior, or body weight (e.g., diabetes), pregnancy, breastfeeding, use of psychotropic medications or substances within the 4 weeks preceding the study. See Supplement A.1 for a full list of exclusion criteria.

All data were managed using the secure, web-based electronic data capture tool REDCap (Research Electronic Data Capture; [26]). This study was approved by the ethics committee of the Technische Universität Dresden and carried out in accordance with the latest version of the Declaration of Helsinki, and all participants (and their guardians if underage) gave written informed consent.

### Clinical measures

For all participants, the presence or absence of current diagnoses of eating disorders were ascertained by evaluation of the expert form of the Structured interview of anorexia nervosa and bulimia nervosa (SIAB-EX; [27]). Additionally, an in-house interview and a structured clinical interview (Mini-DIPS; [28]) were used to assess other psychiatric disorders. Interviews were conducted by clinically experienced and trained research assistants under the supervision of the attending child and adolescent psychiatrist.

In addition to the clinical interviews, eating disorder-specific psychopathology was assessed with the German version of the Eating Disorders Inventory (EDI-2; [29]). Depressive symptoms were examined using the Beck Depression Inventory-II (BDI-II; [30]). IQ was estimated with a short version of the German adaption of the Wechsler Adult Intelligence Scale [31]. As an indicator for weight-to-height ratio that is corrected for age and gender, BMI deviation scores were calculated (BMI-SDS; [25]; see Supplement A.3 for details).

Finally, anxiety and mood were assessed using the German versions of the State Anxiety Inventory (STAI-S; [32]) and Multidimensional Mood State Questionnaire (MDMQ; *Mehrdimensionaler Befindlichkeitsfragebogen, MDBF*; [33]). The STAI-S comprises 20 questions that are summarized to one global anxiety scale with possible scores ranging from 20 (no anxiety) to 80 (extreme anxiety). The MDBF consists of 24 questions that yield three bipolar scales, each scoring from 8 to 40 with higher scores indicating a positive state: bad–good mood, tired–awake, and restless–calm.

### Study protocol

A double-blind, randomized cross-over design with each participant undergoing both ATD and the control condition was employed. Both conditions were separated by a period of 7–14 days to avoid potential carry-over effects (*M* = 9.64, SD = 3.21). On one appointment, the participants took a specific mixture of AAs (see Supplement A.4) that has been shown to induce depletion of TRP supply in the brain (ATD condition). On the other appointment, they took a control mixture that is expected to have no relevant effect on the brain tryptophan supply (control condition) [34].

On the day prior to the study appointments, participants were asked to restrict their meals to food that is low in protein and they were given a list of meal suggestions. On both days, participants arrived at the study center at 7:45 a.m. after an overnight fast and baseline measures were taken; weight and height were measured and plasma was extracted from venous blood. Baseline anxiety and mood were assessed at 8:25 a.m. After the intake of the mixture at 8:30 a.m., participants were given a TRP-free breakfast. Anxiety and mood levels were assessed another five times in 1-h intervals from baseline. At 1:30 p.m., blood for plasma isolation was drawn a second time (follow-up measurement). Besides the standardized breakfast and the ATD or control mixture, the participants did not consume any food and only drank water. For further details regarding the study protocol, see supplement A.5.

### Biochemical measures

To quantify the depletion effect, the ratio of plasma tryptophan (TRP)-to-large neutral amino acids (LNAAs; namely valine, methionine, isoleucine, leucine, phenylalanine, and thyrosine) was calculated (TRP/LNAA ratio) for each participant using the values of the blood samples at baseline and follow-up. The TRP/LNAA ratio is seen as a reliable indicator for the diffusion of tryptophan across the blood–brain barrier and the following production of 5-HT [35]. Supplement A.6 illustrates the preparation and analysis of blood samples and the determination of amino acid levels.

### Statistical analysis

Group differences for demographic variables were assessed with independent samples t tests. In case of a violation of test assumptions, nonparametric tests were performed to confirm the results of parametric testing. Missing data for anxiety and mood questionnaires was 0.4%. For the blood plasma levels of amino acids, 6.4% of the data were missing, resulting from failed blood draw (10 measurements concerning 5 participants) and incorrect handling of probes (one measurement). Missing questionnaire data were imputed with IBM SPSS 25 using the EM algorithm [36].

A two-way repeated-measurements ANOVA was performed to assess the effect of the ATD and control condition on the TRP/LNAA ratio. Subsequently, we aimed to test our main hypothesis that the ATD procedure affects anxiety and mood differently in recAN compared to HC. Following Pruessner et al. [37], we computed for each participant and condition (ATD/control) an area under the curve of the hourly measured STAI-S and MDBF questionnaires subtracted by the corresponding baseline value (AUC_I,_ see Supplement A.7). The four AUC_I_ were subjected to repeated-measures ANOVAs, with group as a between-subjects factor, condition as a within-subjects factor, and order of randomization as a covariate. In this model, a significant group*condition interaction would provide evidence in favor of the 5-HT hypothesis. Following Kaye et al. [21], we also conducted simple paired t-tests to assess the effect of conditions for each scale separately within each group. Furthermore, we planned to conduct Bayesian analyses in case of non-significant results to assess to which extent these provide support for the null hypothesis in contrast to being due to lack of statistical power. We corrected for multiple testing using the Bonferroni method. SPSS 25 and JASP 0.13.1 [38] were used for the data analysis.

## Results

### Sample description and ATD procedure

Demographic and clinical characteristics are summarized in Table 1. As expected, recAN and HC participants differed in minimum lifetime BMI and EDI-2. The groups did not differ significantly in age, current BMI-SDS, IQ, days since last menorrhea, or depressive symptoms.

**Table 1:** Demographics and selected clinical characteristics of sample

	recAN (*n* = 22)	HC (*n* = 25)	*T* test		
			*T*	*df*	*P*
Age [years]	23.00 ± 2.80	23.00 ± 2.50	0.08	45	0.935
BMI [kg/m^2^]	21.03 ± 1.48	21.06 ± 2.02	− 0.05	45	0.959
BMI-SDS	− 0.43 ± 0.52	− 0.46 ± 0.63	0.18	45	0.858
BMI minima [kg/m^2^]	14.51 ± 1.50	19.64 ± 1.25	− 12.6	44	< 0.001
IQ	111 ± 8	112 ± 8	− 0.17	45	0.863
Days since last menses	18 ± 19	16 ± 13	0.30	42	0.765
Recovered since months	58 ± 34	n/a	n/a	n/a	n/a
BDI-II	6.18 ± 8.19	2.44 ± 3.27	2.01	26.83*	0.055
EDI-2 total	20.23 ± 5.77	15.56 ± 2.18	3.58	26.24*	0.001

Data are presented as mean ± standard deviation. Twenty women had a history of restrictive type AN, one of binge/purge type AN, and one of restrictive type with intermittent binge/purge behavior. Three recAN had comorbidities at the time of treatment: two had depressive disorders and one had an anxiety disorder. Group differences are assessed using independent t tests

*BDI*-*2* Beck depression inventory-2, *BMI* body mass index, *BMI* minima/maxima lifetime minimum/maximum in BMI, *IQ* intelligence quotient, *EDI*-*2* eating disorder inventory-2, *HC* healthy control women, *JCTI* junior temperament and character inventory (only two out of seven subscales are displayed), *recAN* recovered former anorexia nervosa patients

*Greenhouse–Geisser corrected degree of freedom due to inequality of variances

A highly significant interaction between the experimental condition (ATD vs. control condition) and time of measurement (before vs. after procedure) for the TRP/LNAA ratio was revealed, thus confirming the desired differential effect of the two experimental conditions on the TRP/LNAA ratio (*F* = 295.2, df = 1, *p* < 0.001; Table S1, see Figure S1 for separate group values).

### STAI-S and MDBF

In a repeated-measures ANOVA, neither the main effect of group nor the interaction group*condition was significant after correcting for multiple testing (Table 2, see Figure S2 for AUC_I_ values for each individual scale; for detailed descriptive values, see Table S2). Follow-up sensitivity analyses confirmed similar results for a sample including only restrictive type AN patients (Table S3) and before imputation of missing values (Table S4). However, the main effect of condition showed a statistical trend in the latter two analyses, i.e., participants were less tired under the ATD condition (Tables 2, S3, S4). Similarly, despite sufficient statistical power as suggested by the G*Power analysis, paired t tests showed no significant effect on condition for the STAI scale and the MDBF subscales‚ ‘bad-good mood’ and ‘nervous-calm’ for both groups. On the ‘tired-awake’ scale recAN participants scored significantly higher on the ATD day (*T*(21) = − 3.032, *p* = 0.006, Bonferroni-corrected α = 0.0125), while for HC participants, this was true at the trend level (*T*(24) = − 2.072, *p* = 0.049; Table S5).

**Table 2:** Anxiety and Mood State changes after intake of ATD or control mixture

				Two-way ANOVA								
	Group	Control	ATD	Group			Condition			Group*cond		
				*F*	*df*	*P*	*F*	*df*	*p*	*F*	*df*	*P*
Anxiety (STAI-S)	recAN	1.15 ± 2.50	0.29 ± 3.26	0.00	1	0.954	0.36	1	0.550	0.01	1	0.909
	HC	1.45 ± 4.61	0.53 ± 3.14									
Bad–good mood (MDBF)	recAN	− 1.90 ± 2.92	− 1.28 ± 3.14	0.07	1	0.799	3.94	1	0.054	1.95	1	0.170
	HC	− 1.94 ± 3.22	− 0.49 ± 1.65									
Tired–awake (MDBF)	recAN	− 2.70 ± 3.65	− 0.08 ± 3.90	0.01	1	0.937	6.09	1	0.018	0.17	1	0.683
	HC	− 2.64 ± 4.97	− 0.27 ± 2.88									
Nervous–calm (MDBF)	recAN	0.06 ± 2.44	− 0.03 ± 2.84	2.38	1	0.130	1.13	1	0.294	0.00	1	0.989
	HC	− 1.03 ± 3.60	− 0.59 ± 2.59									

Data is presented as mean ± standard deviation for AUC_I_; for descriptive values of each single time point, see Supplement B.4. Significance testing was carried out with a repeated-measures analysis of variance with ATD vs. control condition as within-subjects factor, group as between-subjects factor, and order of randomization as covariate. Positive STAI-S scores represent an increase from baseline and consequently a worsening of symptoms (higher anxiety), while positive MDBF scores represent an improvement of symptoms (better mood, more awake, and more calm). Mean anxiety and mood state scores for each single time point are reported in Table S4. Changes were computed as area under the curve of the hourly STAI-S and MDBF questionnaires scales subtracted by their individual baseline (AUC_I_). The Bonferroni corrected significance level was α = 0.0125

*HC* healthy control women, *MDBF* Mehrdimensionaler Befindlichkeitsfragebogen (German version of multidimensional mood state questionnaire, MDMQ), *recAN* recovered former anorexia nervosa patients, *STAI*-*S* state-trait anxiety inventory, state anxiety score

Bayesian analysis of repeated-measures ANOVAs revealed that data are approximately 2.5 to 3.4 times more likely to occur under H0 than under H1 (Table 3). This result indicates anecdotal-to-moderate evidence in favor of H0, i.e., the absence of an interaction effect [39]. Bayes factors for each paired samples *t* test (as originally employed in the analysis by Kaye et al. [21]) indicated anecdotal-to-moderate evidence that ATD did not improve self-reported anxiety and mood in recAN, except for the tired/awake scale, where our analysis suggested moderate and anecdotal evidence for the alternative hypothesis in recAN and HC, respectively (for both in the same direction, Table S6).

**Table 3:** Results of Bayesian repeated-measurements ANOVA testing for a group*condition interaction for AUC increase of hourly STAI-S and MDBF questionnaires scores

		P(M|data)	BF_M_	BF_01_
Anxiety (STAI-S)	H0	0.761	3.186	1
	H1	0.239	0.314	3.186
Bad–good mood (MDBF)	H0	0.716	2.526	1
	H1	0.284	0.396	2.526
Tired–awake (MDBF)	H0	0.771	3.376	1
	H1	0.229	0.296	3.376
Nervous–calm (MDBF)	H0	0.750	3.002	1
	H1	0.250	0.333	3.002

The null hypothesis model (H0) presumes that group (recAN vs. HC) and condition (ATD vs. control) predict the questionnaire scores. The alternative hypothesis model (H1) postulates that the additional inclusion of the interaction effect group*condition improves the predictive accuracy. P(M|data) estimates the probability that the respective model is correct (relative to the other model) assuming these data. BF_M_ displays the relative predictive accuracy of one model against the other model, with values larger than 1 indicating a preference for the current model (e.g., the H0 model is approx. 3.2 times more probable to explain the STAI-S score than the H1 model). The Bayes factor BF_01_ quantifies the relative predictive accuracy of the H0 model over the H1 model, with values larger than 1 indicating a preference for H0 (e.g., the H1 model is approx. 3.2 less likely to explain the STAI-S score than the H0 model)

*MDBF* *Mehrdimensionaler Befindlichkeitsfragebogen* (German version of multidimensional mood state questionnaire, MDMQ), *STAI*-*S* state-trait anxiety inventory, state anxiety score

## Discussion

In this study, we aimed to gather evidence for or against the 5-HT hypothesis of AN by attempting to reproduce the results from a previous study suggesting that a dietary-induced depletion of the 5-HT precursor tryptophan (ATD) has anxiolytic effects in individuals recovered from AN, but not HC [21] and by investigating its effect on mood. However, in our study, the ATD procedure did not induce any significant differential change in self-reported anxiety or mood in recAN vs. HC participants. In fact, a subsequent Bayesian analysis provided moderate evidence in favor of the null hypothesis. In line with most previous literature [40, 41] and our hypothesis for this study, our analysis further suggests that ATD has no significant effect on anxiety or mood in HC individuals.

The ATD procedure successfully reduced plasma levels of both tryptophan and the TRP/LNAA ratio by more than 60% (Figure S1), a threshold above which effects on mood were detected [42]. In contrast to Kaye et al. [21], we did not find an improvement in anxiety in the ATD condition for recAN. This could be due to deviations in study design; for instance, the instruments used to assess anxiety, the presence of a smaller percentage of participants that had suffered from the binge/purge subtype, or a longer time since weight recovery compared to Kaye et al. [21]. However, similar to the results of both our frequentist and Bayesian analyses, even in the original Kaye et al.’s [21] publication, the critical group*condition interaction was not significant. Together, these findings are not in line with the 5HT-hypothesis of AN, which predicts that ATD would have a differential effect on anxiety in recAN and HC, by balancing 5-HT availability in recAN, but by reducing it to suboptimal levels in HC.

While our results do not indicate that ATD improves anxiety in recAN, it is difficult to draw firm conclusions regarding the premorbid state or individuals at risk of AN. Although there is cumulative evidence for alterations in the 5-HT system in AN [10, 16, 18, 43], it is not clear exactly when this alteration manifests. As AN is a comparably rare condition, hardly any neurobiological data from premorbid individuals is available. In the acute phase, the distorted metabolic situation prevents the discrimination between cause and effect. In weight-recovered individuals, both long-term consequences (‘scarring’) and a premorbid trait seem plausible to explain a persistent alteration in the 5-HT system [3, 44]. Twin studies that observe the diseased twin and the monozygotic healthy twin [44] could give important insights into whether the alteration in 5-HT activity is a premorbid trait marker or a scar.

If a premorbid 5-HT dysregulation in AN is nevertheless conditionally accepted, it is essential to empirically support its assumed causal link to anxiety and mood to support the 5-HT hypothesis of AN. To our knowledge, only Kaye et al. [21] experimentally tested the effects of TRP availability on anxiety and mood in recAN so far. Another study focused on acute AN patients, which are assumed to be characterized by depleted 5-HT availability [14]. [10]. In this study, acute AN patients received either TRP or a placebo together with the selective serotonin reuptake inhibitor (SSRI) fluoxetine, but no effects of the supplementation on questionnaires measuring anxiety and obsessive–compulsive symptoms were found [45]. Non-experimental studies found decreased anxiety and depressive symptoms in the course of re-feeding [11, 46]. In these non-controlled studies, however, many factors other than 5-HT availability may have contributed to the improvement in symptoms that were observed during re-feeding.

Various affective and cognitive states have been attributed to either high or low 5-HT activity [40, 47–49]. Notably, anxiety has been associated with both high and low 5-HT activity: while some studies find that a reduction in 5-HT neuromodulation reduces anxiety and a stimulation increases anxiety in animal models [50] and that anxiety in patients with a social anxiety disorder (SAD) is associated with increased 5-HT synthesis and transporter availability [51], other studies find that ATD slightly elevates anxiety in patients with SAD or individuals recovered from SAD or panic disorder [40] and that SSRIs, which increase extracellular 5-HT, work well in patients with SAD and obsessive–compulsive disorder (OCD) [52–54]. This evidence does not suggest a stable, linear relationship between 5-HT levels and anxiety, but rather indicates an inverted U-shaped curve with optimal psychological states being statistically associated with average 5-HT activity. If patients with AN indeed show excessive 5-HT activity before the onset of the eating disorder and if altered dietary intake can moderate this, anxiety and possibly other psychological states might improve when their 5-HT system can be modulated to fluctuate in a more balanced range. As for now, however, more evidence in favor of these two aspects is needed.

Some limitations to this study deserve special mention. First, we only used self-report measures to assess anxiety and mood; alternative dependent measures, such as implicit measures, expert ratings, or neurophysiological parameters, should be considered. Second, ethical reasons prevented us from also including acutely underweight patients with AN in our sample as previous studies in patients with bulimia nervosa [55, 56] and depression [57] suggested that the ATD procedure might cause a relapse or exacerbate symptoms in some patients [58]. Third, there were not enough recAN participants of previous binge/purge subtype AN in this study to accomplish meaningful results for this group of patients.

In summary, our results do not support the previous finding that a dietary lowering of tryptophan levels has differential effects on self-rated anxiety in former AN patients vs. HC individuals. Given the need for a more detailed understanding of the neurobiology of AN and the potential of the 5-HT hypothesis to pave the way for the development of much-needed pharmacological interventions specific to AN, studies based on other methodologies to gather more evidence in favor or against this hypothesis are needed. Twin studies comparing the affected and the monozygotic healthy twin [44] could give important insights into whether the alteration in 5-HT activity is a premorbid trait marker or a scar.

## Supplementary Information

Below is the link to the electronic supplementary material.

Supplementary file1 (DOCX 289 KB)
