# Supplementary material for: No effects of acute tryptophan depletion on anxiety or mood in weight-recovered female patients with anorexia nervosa
Source: Eur Arch Psychiatry Clin Neurosci. 2022 May 5;273(1):209–17. doi: 10.1007/s00406-022-01414-8 (PMC9957824; doi:10.1007/s00406-022-01414-8)
Supplement: Supplementary file 1 — Supplementary file1 (DOCX 289 KB) [file 406_2022_1414_MOESM1_ESM.docx]

**No effects of acute tryptophan depletion on anxiety or mood in recovered female patients with anorexia nervosa**

**Supplementary materials**

## A Methods

### A.1 Full list of inclusion- and exclusion criteria

As in our previous studies that focused on weight-recovered individuals with a history of anorexia nervosa (recAN) [1,2], participants of both groups were excluded if they reported current selective serotonin reuptake inhibitors (SSRI) intake, psychotropic drug intake within the last four weeks, being pregnant or breast feeding, anemia, being younger than 12 years or 30 years and older, having an intelligence quotient (IQ) below 85, suffering from organic brain syndrome, dementia, schizophrenia, bipolar disorder, drug abuse, obesity (BMI >97th percentile for age <18, BMI >30 for age 18 and older) as well as chronic medical or neurological illnesses that could affect appetite, eating behavior or body weight (e.g. diabetes).

As for the healthy control participants (HC), they were excluded if they had a lifetime diagnosis of any psychiatric disorder, the lowest lifetime BMI below the 10^th^ percentile (below age 18) or <17.5 (for age 18 and older) or if they were currently underweight, showed abnormal eating behavior (diet, binge eating) or a binge eating disorder.

Additionally, recAN were excluded if they had a lifetime diagnosis of atypical AN, bulimia nervosa or binge eating disorder.

### A.2 In-house semi-structured Interview

Our in-house interview that has been already employed in our previous studies on AN [1,1,3,4]. The goal of this procedure is a comprehensive description of our participant groups. Using the semi-structured interview, demographic and clinical data in the following domains are assessed by trained psychologists and medical students: Socio-economic status, family history, detailed information on body weight, including highest and lowest lifetime-BMI and weight changes over the past six weeks, detailed information on menstrual cycle and contraceptives intake, current and past medical problems, current and past psychiatric or psychological treatments, current and past medication intake and nicotine abuse.

### A.3 Calculation of BMI-SDS values

BMI-SDS scores were calculated according to the method by Kromeyer-Hauschild et al. [5] to provide an index of weight to height ratio that is corrected for age and gender.

### A.4 Composition of ATD and control mixture

The method was originally proposed by Moja et al. [6] and adjusted to children and adolescents by Zepf et al. [7]. See also Stewart et al. [8] for a comprehensive description of the procedure and molecular details of ingredients. In our study, the two mixtures contain the following amino acids:

- L-Phenylalanine (PHE), 1.32 g/10 kg of body weight
- L-Leucine (LEU), 1.32 g/10 kg of body weight
- L-Isoleucine (ILE), 0.84 g/10 kg of body weight
- L-Methionine (MET), 0.5 g/10 kg of body weight
- L-Valine (VAL), 0.96 g/10 kg of body weight
- L-Threonine (THR), 0.6 g/10 kg of body weight
- L-Lysine (LYS), 0.96 g/10 kg of body weight
- Only in control mixture: L-Tryptophan (TRP), 0.7 g/10 kg of body weight

The experimental mixture for each participant was prepared according to the proportions mentioned above with material that was purchased from the associated pharmacy of the University Hospital Carl Gustav Carus in Dresden, Germany. A base mixture for 45kg of body weight was prepared; depending on the actual weight of the participant, additional sachets that corresponded to 5kg each were added. E.g., a participant with 56.5 kg of body weight falls into the weight interval of 52.5kg ≤ body weight < 57.5kg and therefore received the base mixture (corresponding to 45 kg of body weight) and additional two sachets (corresponding to 5 kg of body weight, respectively). The appropriate amount of powder was dissolved in approximately 300 ml of Sprite Zero ® lemonade.

### A.5 Study protocol

On both days, participants arrived at the study center at 7:45 a.m. after an overnight fast and venous blood was drawn into tubes for extraction of blood plasma. At 8:25 a.m., anxiety and mood were assessed using the German versions of the State Anxiety Inventory (STAI-S) [9,10] and Multidimensional Mood State Questionnaire (MDMQ; *Mehrdimensionaler Befindlichkeitsfragebogen, MDBF*) [11]. Both instruments are frequently employed in psychiatry research and exhibit good reliability and validity [9,12].

After the intake of the mixture at 8:30 a.m., participants were given a tryptophan free breakfast. Anxiety and mood levels were assessed another five times in one-hour intervals from baseline (i.e. at 9:25 a.m., 10:25 a.m., 11:25 a.m., 12:25 p.m., 1:25 p.m.). At 1:30 p.m., 5:45 hours after the baseline measurement, blood for plasma isolation was drawn a second time (follow-up measurement). Besides the standardized breakfast and the ATD or control mixture, the participants did not consume any food and only drank water. Participants were continuously supervised by trained staff.

### A.6 Blood sample analysis

To determine amino acid levels, venous blood was drawn into tubes for extraction of blood plasma (tubes by Sarstedt, Germany, containing ethylenediaminetetraacetic (EDTA), 1.6 mg/ml, and Aprotinin, 270 KIU/ml). Immediately after taking the blood samples, they were centrifuged at 4,000g and 4°C for 10 minutes. Afterwards, the plasma was stored in a -81°C fridge. Finally, blood analyses were conducted at the Institute for Clinical Chemistry and Laboratory Medicine of the Technische Universität Dresden, Medizinische Fakultät. The analyses of physiological amino acids were performed by cation exchange chromatography with post-column derivatization, using the Biochrome amino acid analyzer B30. To avoid influences of calibration, all samples from each patient were run within one and the same series, using additional internal standard in each sample.

### A.7 Computation of AUC

The following formula was used to calculate the AUC_I_ values (Area under the curve with respect to increase) presented in this study:

$${AUC}_{I}=\frac{\left( \sum_{i=1}^{n-1} \frac{(m_{\left( i+1 \right)}+m_{i})}{2} \right)}{n-1}-m_{1}$$

where:

$m_{i}$ = single measurement

$n$ = total number of measurements

The abovementioned formula calculates the increase in AUC and keeps the resulting values within the scoring range of the respective questionnaire by dividing the sum by the number of measurement intervals. We did this to facilitate interpretation of the extent of changes regarding the content of the questionnaire. It was derived from the following formula proposed by Pruessner et al. [13] (formula 6):

$${AUC}_{I}=\left( \sum_{i=1}^{n-1} \frac{(m_{\left( i+1 \right)}+m_{i})}{2} \right)-\left( n-1 \right)*m_{1}$$

## B Supplementary Results

### B.1 Extended lab results

**Table S1. TRP/LNAA ratio according to amino acid levels in the blood plasma for baseline and follow-up measure.**

|  | TRP/LNAA ratio | | two-way rmANOVA | | | | | | | | |
| --- | --- | --- | --- | --- | --- | --- | --- | --- | --- | --- | --- |
|  |  |  | *condition* | | | *time* | | | *condition*time* | | |
| Condition | Baseline | Follow-up | *F* | *df* | *p* | *F* | *df* | *p* | *F* | *df* | *p* |
| Control (n = 44) | 0.11 ± 0.02 | 0.19 ± 0.07 | 165.3 | 1 | <.001 | 1.08 | 1 | .302 | 295.2 | 1 | <.001 |
| ATD (n = 41) | 0.10 ± 0.02 | 0.01 ± 0.01 |  |  |  |  |  |  |  |  |  |

ATD = acute tryptophan depletion; condition = ATD or control condition; rmANOVA = repeated measurements Analysis of covariance; time = blood sample before vs. after ATD/control drink; TRP/LNAA ratio = ratio of tryptophan to large neutral amino acids that compete with tryptophan for transportation across the blood brain barrier (includes isoleucine, leucine, phenylalanine, tyrosine, valine, and methionine). Data is presented as mean ± standard deviation. The follow-up measure was taken 5:15 hours after application of ATD or control mixture.

### B.2 Extended results for mood ratings

**Table S2. Mean anxiety and mood state scores for each single time point.**

|  | **Group** | **Condition** | **T1** | **T2** | **T3** | **T4** | **T5** | **T6** |
| --- | --- | --- | --- | --- | --- | --- | --- | --- |
| STAI-S Anxiety | HC | Control | 31.04±6.85 | 33.12±8.05 | 33.80±9.32 | 32.60±8.78 | 32.76±7.65 | 29.27±6.31 |
|  |  | ATD | 30.08±6.28 | 31.32±7.49 | 31.28±7.80 | 30.24±6.90 | 30.64±8.46 | 29.08±6.57 |
|  | recAN | Control | 36.68±10.27 | 37.50±8.51 | 38.61±9.86 | 38.27±9.80 | 38.36±11.01 | 36.18±9.08 |
|  |  | ATD | 36.55±8.32 | 38.00±8.80 | 37.50±9.30 | 36.64±9.59 | 36.14±9.16 | 35.64±8.98 |
| MDBF  Bad – good  mood | HC | Control | 36.40±3.61 | 33.84±5.41 | 32.60±5.74 | 34.76±5.03 | 35.36±3.90 | 35.08±4.85 |
|  |  | ATD | 36.60±2.68 | 35.12±4.02 | 35.56±4.09 | 36.44±3.40 | 36.72±3.70 | 36.84±3.39 |
|  | recAN | Control | 31.91±5.01 | 29.59±6.71 | 28.86±6.03 | 30.00±5.98 | 30.23±6.26 | 30.86±7.45 |
|  |  | ATD | 33.91±4.34 | 30.41±5.75 | 31.95±4.65 | 32.64±4.34 | 34.05±4.04 | 33.77±4.05 |
| MDBF  Tired – alert | HC | Control | 31.24±4.08 | 28.44±5.73 | 25.48±8.19 | 27.56±8.52 | 30.64±5.77 | 30.51±6.07 |
|  |  | ATD | 31.72±5.28 | 31.76±7.16 | 30.00±6.61 | 31.32±5.44 | 32.88±5.73 | 30.84±7.52 |
|  | recAN | Control | 26.41±6.91 | 23.86±5.94 | 22.23±7.50 | 21.77±5.55 | 25.32±6.46 | 24.27±7.39 |
|  |  | ATD | 26.36±7.09 | 25.50±7.37 | 25.59±7.59 | 25.41±6.91 | 28.18±6.56 | 27.05±7.38 |
| MDBF  Nervous – calm | HC | Control | 35.60±3.16 | 34.24±5.25 | 33.76±5.55 | 34.72±5.81 | 34.56±4.81 | 35.53±5.03 |
|  |  | ATD | 36.12±3.27 | 35.56±3.92 | 35.28±4.71 | 35.44±4.36 | 35.28±5.95 | 36.04±4.26 |
|  | recAN | Control | 31.68±6.51 | 32.14±5.98 | 31.32±6.27 | 31.73±5.58 | 31.77±6.22 | 31.82±7.31 |
|  |  | ATD | 33.27±3.65 | 32.50±5.04 | 32.45±5.40 | 33.27±5.02 | 33.86±5.06 | 34.45±4.01 |
| ATD = acute tryptophan depletion; HC = healthy control women; MDBF = Mehrdimensionaler Befindlichkeitsfragebogen (German version of Multidimensional Mood State Questionnaire, MDMQ); recAN = recovered former anorexia nervosa patients; STAI-S = State-Trait Anxiety Inventory, state anxiety score. Data is presented as mean±standard deviation. | | | | | | | | |

**B.3 Sensitivity analyses**

| **Table S3. Test results of repeated measurements ANOVA for Anxiety and Mood State changes in AUC after intake of ATD or control mixture *without binge/purge subtype AN*.** Changes were computed as area under the curve of the hourly STAI-S and MDBF questionnaires scales subtracted by their individual baseline (AUC_I_). The Bonferroni corrected significance level was α = 0.0125. | | | | | | | | | | |
| --- | --- | --- | --- | --- | --- | --- | --- | --- | --- | --- |
|  | **ANOVA** | | | | | | | | | |
|  | *group* | | | *condition* | | | *group*cond.* | | | |
|  | *F* | *df* | *p* | *F* | *df* | *p* | *F* | *df* | *P* |  |
| Anxiety (STAI-S) | 0.186 | 1 | .668 | 0.308 | 1 | .582 | 0.004 | 1 | .948 |  |
|  |  |  |  |  |  |  |  |  |  |  |
| Bad – good mood (MDBF) | 0.00 | 1 | .989 | 3.347 | 1 | .074 | 1.389 | 1 | .245 |  |
|  |  |  |  |  |  |  |  |  |  |  |
| Tired – awake (MDBF) | 0.194 | 1 | .662 | 7.155 | 1 | .011 | 0.569 | 1 | .455 |  |
|  |  |  |  |  |  |  |  |  |  |  |
| Nervous – calm (MDBF) | 3.241 | 1 | .079 | 0.965 | 1 | .332 | 0.034 | 1 | .855 |  |
|  |  |  |  |  |  |  |  |  |  |  |
| MDBF = Mehrdimensionaler Befindlichkeitsfragebogen (German version of Multidimensional Mood State Questionnaire, MDMQ); STAI-S = State-Trait Anxiety Inventory, state anxiety score. Data is presented as mean±standard deviation for AUC_I_. Significance testing was carried out with a repeated measures analysis of variance with ATD vs. control condition as within-subjects factor, group as between-subjects factor, and order of randomization as covariate. | | | | | | | | | |  |

**Table S4. Test results of repeated measurements ANOVA for Anxiety and Mood State changes in AUC after intake of ATD or control mixture *without imputation of missing values*.** Changes were computed as area under the curve of the hourly STAI-S and MDBF questionnaires scales subtracted by their individual baseline (AUC_I_). The Bonferroni corrected significance level was α = 0.0125.

|  | **ANOVA** | | | | | | | | | |
| --- | --- | --- | --- | --- | --- | --- | --- | --- | --- | --- |
|  | *group* | | | *condition* | | | *group*cond.* | | | |
|  | *F* | *df* | *p* | *F* | *df* | *p* | *F* | *df* | *P* |  |
| Anxiety (STAI-S) | .032 | 1 | .859 | .430 | 1 | .515 | .000 | 1 | .987 |  |
|  |  |  |  |  |  |  |  |  |  |  |
| Bad – good mood (MDBF) | .423 | 1 | .519 | 5.648 | 1 | .022 | 1.370 | 1 | .248 |  |
|  |  |  |  |  |  |  |  |  |  |  |
| Tired – awake (MDBF) | .062 | 1 | .805 | 7.272 | 1 | .010 | .586 | 1 | .448 |  |
|  |  |  |  |  |  |  |  |  |  |  |
| Nervous – calm (MDBF) | 2.247 | 1 | .141 | .634 | 1 | .430 | .042 | 1 | .839 |  |
|  |  |  |  |  |  |  |  |  |  |  |

MDBF = Mehrdimensionaler Befindlichkeitsfragebogen (German version of Multidimensional Mood State Questionnaire, MDMQ); STAI-S = State-Trait Anxiety Inventory, state anxiety score. Significance testing was carried out with a repeated measures analysis of variance with ATD vs. control condition as within-subjects factor, group as between-subjects factor, and order of randomization as covariate.

### B.4 Supplementary inferential statistics

**Table S5.** **Anxiety and Mood State changes after intake of ATD or control mixture and results of associated dependent t-tests.** Changes were computed as area under the curve of the hourly STAI-S and MDBF questionnaires scales subtracted by their individual baseline (AUC_I_). The Bonferroni corrected significance level was α = 0.0125.

|  |  |  |  | **t-test** | | |
| --- | --- | --- | --- | --- | --- | --- |
|  | Group | Control | ATD | *T* | *df* | *P* |
| Anxiety (STAI-S) | recAN | 1.15±2.50 | 0.29±3.26 | 1.187 | 21 | .249 |
|  | HC | 1.45±4.61 | 0.53±3.14 | 1.007 | 24 | .324 |
| Bad – good mood (MDBF) | recAN | -1.90±2.92 | -1.28±3.14 | -1.116 | 21 | .277 |
|  | HC | -1.94±3.22 | -0.49±1.65 | -2.128 | 24 | .044 |
| Tired – awake (MDBF) | recAN | -2.70±3.65 | -0.08±3.90 | -3.032 | 21 | .006 |
|  | HC | -2.64±4.97 | -0.27±2.88 | -2.072 | 24 | .049 |
| Nervous – Calm  (MDBF) | recAN | 0.06±2.44 | -0.03±2.84 | 0.102 | 21 | .920 |
|  | HC | -1.03±3.60 | -0.59±2.59 | -0.602 | 24 | .553 |

HC = healthy control women; MDBF = *Mehrdimensionaler Befindlichkeitsfragebogen* (German version of Multidimensional Mood State Questionnaire, MDMQ); recAN = recovered former anorexia nervosa patients. STAI-S = State-Trait Anxiety Inventory, state anxiety score. Data is presented as mean±standard deviation. Dependent t-tests (ATD vs. control condition) were calculated for each group separately.

### B.5 Supplementary Bayesian analysis

In addition to the Bayesian analysis presented in the results part, we performed Bayesian paired samples t-tests to evaluate whether the condition (ATD vs. control) had an effect on any of the four scales when recAN and HC participants are considered separately. These tests were performed to mirror the tests employed by Kaye et al. [14] in the original study. Results are displayed in table S4.

**Table S6. Bayes factors (BF_01_) presenting the evidence for the null hypothesis (H0) from Bayesian dependent t-tests for AUC increase of hourly STAI-S and MDBF questionnaire scores.** Tests compare ATD vs. control condition separately for each group.

|  | BF_01_ | | | |
| --- | --- | --- | --- | --- |
|  | Anxiety (STAI-S) | Bad / good mood (MDBF) | Tired / awake (MDBF) | Nervous / calm (MDBF) |
| recAN | 2.409  ATD ˂ Control | 2.584  ATD ˃ Control | 0.137  ATD ˃ Control | 4.464  ATD < Control |
| HC | 3.005  ATD ˂ Control | 0.702  ATD ˃ Control | 0.770  ATD ˃ Control | 4.020  ATD > Control |

MDBF = Mehrdimensionaler Befindlichkeitsfragebogen (German version of Multidimensional Mood State Questionnaire, MDMQ); STAI-S = State-Trait Anxiety Inventory, state anxiety score. The null hypothesis (H0) postulates that there is no difference in the AUC increase for the questionnaire scores between conditions. The alternative hypothesis model (H1) postulates that in the ATD condition the AUC increase differs between conditions. The Bayes factor BF_01_ quantifies the relative predictive accuracy of the H0 model over the H1 model, with values larger than 1 indicating a preference for H0. In particular: 1<BF01<3 and 3<BF01<10 indicate anecdotal and moderate evidence for H0, respectively. Instead, 1>BF01>1/3 and 1/3>BF01>1/10 indicate anecdotal and moderate evidence for H1, respectively. Less than/greater than symbols indicate in which condition the absolute AUC_I_ scores are descriptively larger, e.g. ATD < Control indicates that the STAI-S Anxiety score was larger in the control condition.

**Figures**


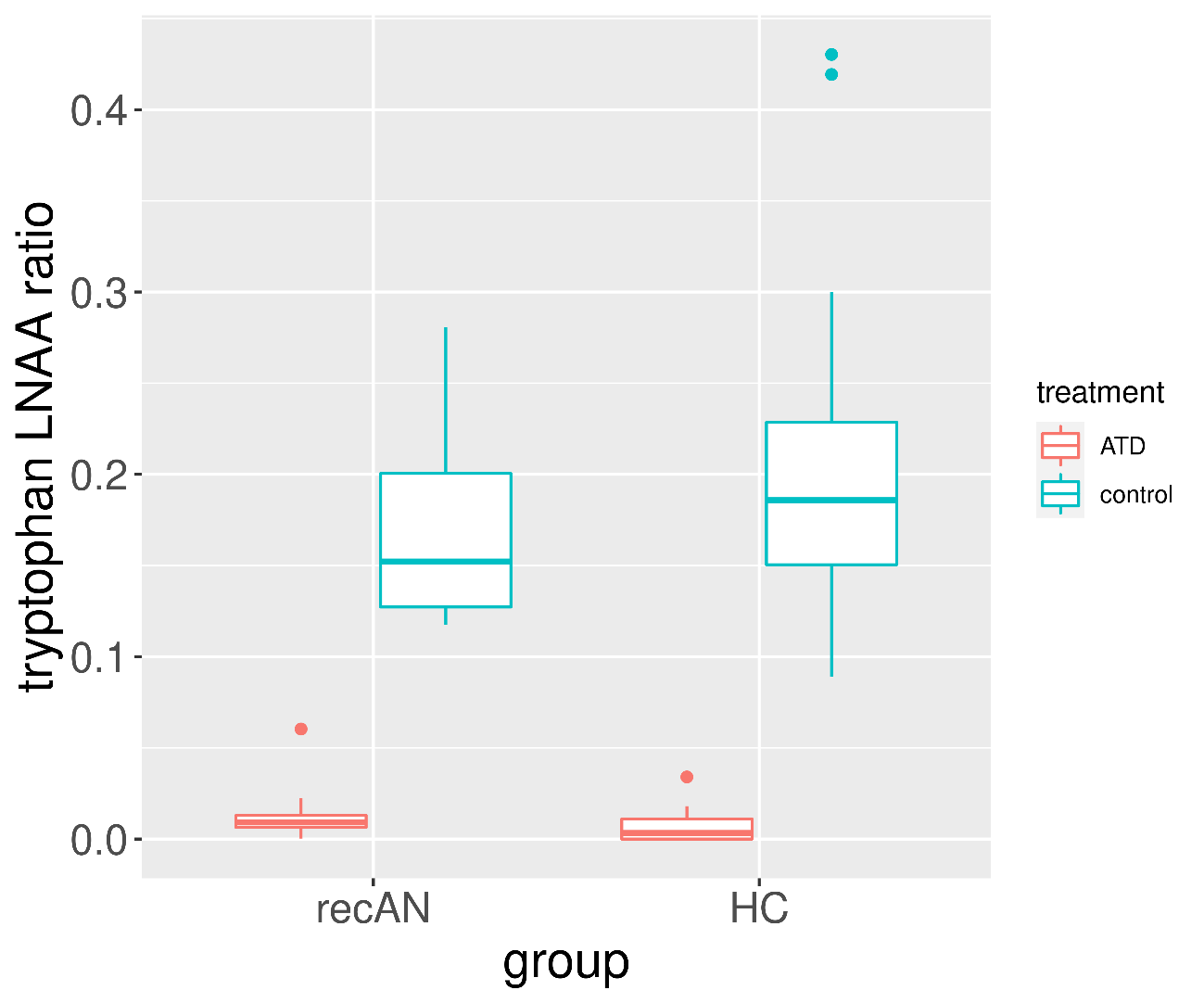


**Figure S1**. Boxplots of plasma tryptophan ratios of each group for both conditions showing the successful treatment effect of the acute tryptophan depletion. After ATD, TRP was reduced by mean(SD)=89(11)% in recAN and mean(SD)=94(8)% in HC. LNAA = long neutral amino acids, recAN = patient recovered from anorexia nervosa, HC = healthy control participant, ATD = acute tryptophan depletion.

**
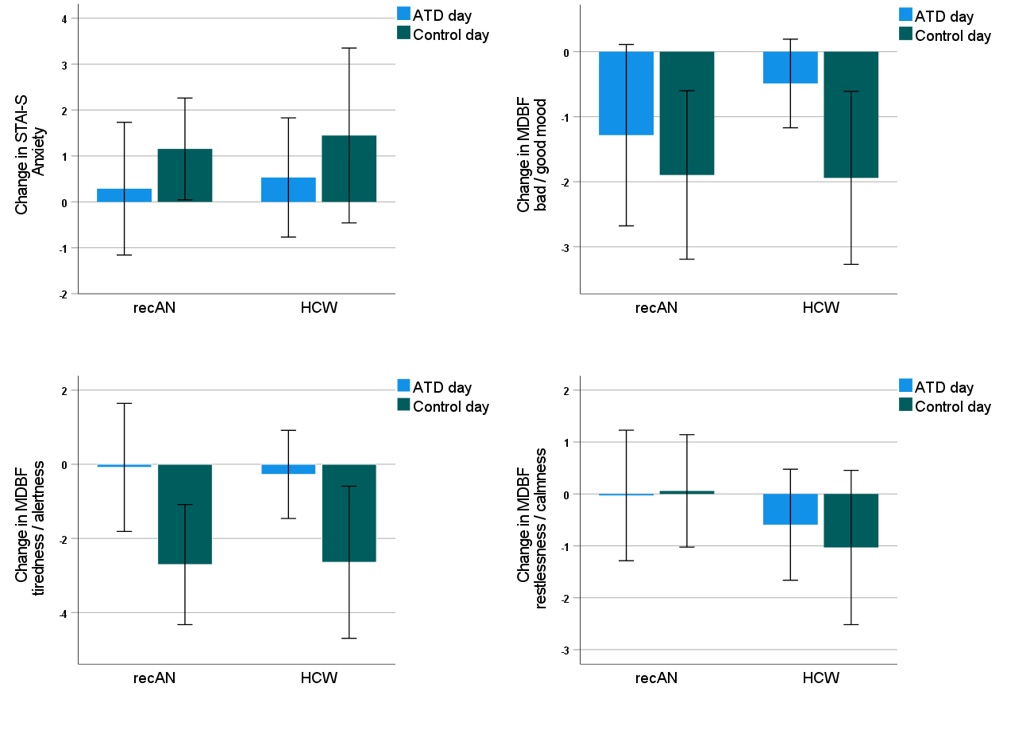
**

**Figure S2.** Mean values and 95 % error bar (confidence interval) for changes in area under the curve for anxiety (STAI-S) and mood state (MDBF) questionnaires for both groups and conditions and for each scale of the questionnaire. Since the AUC was divided by the number of measurement intervals, all scores can be understood as the average change within the usual scoring limits of the respective questionnaire (20 to 80 for STAI-S, 8 to 40 for MDBF).

**Supplemental References**

[1] King JA, Geisler D, Ritschel F, et al. Global Cortical Thinning in Acute Anorexia Nervosa Normalizes Following Long-Term Weight Restoration. Biological Psychiatry 2015; 77: 624–632. doi:10.1016/j.biopsych.2014.09.005

[2] Bernardoni F, Bernhardt N, Pooseh S, et al. Metabolic state and value-based decision-making in acute and recovered female patients with anorexia nervosa. J Psychiatry Neurosci 2020; 9

[3] Boehm I, Geisler D, King JA, et al. Increased resting state functional connectivity in the fronto-parietal and default mode network in anorexia nervosa. Front Behav Neurosci 2014; 8. doi:10.3389/fnbeh.2014.00346

[4] Bernardoni F, King JA, Geisler D, et al. Nutritional Status Affects Cortical Folding: Lessons Learned From Anorexia Nervosa. Biological Psychiatry 2018; 84: 692–701. doi:10.1016/j.biopsych.2018.05.008

[5] Kromeyer-Hauschild K, Wabitsch M, Kunze D, et al. Perzentile für den Body-mass-Index für das Kindes- und Jugendalter unter Heranziehung verschiedener deutscher Stichproben. Monatsschr Kinderheilkd 2001; 149: 807–818. doi:10.1007/s001120170107

[6] Moja EA, Stoff DM, Gessa GL, et al. Decrease in plasma tryptophan after tryptophan-free amino acid mixtures in man. Life Sciences 1988; 42: 1551–1556. doi:10.1016/0024-3205(88)90013-6

[7] Zepf FD, Holtmann M, Stadler C, et al. Diminished central nervous 5-HT neurotransmission and mood self-ratings in children and adolescents with ADHD: no clear effect of rapid tryptophan depletion. Human Psychopharmacology: Clinical and Experimental 2009; 24: 87–94. doi:10.1002/hup.1002

[8] Stewart RM, Wong JWY, Mahfouda S, et al. Acute Tryptophan Depletion Moja-De: A Method to Study Central Nervous Serotonin Function in Children and Adolescents. Front Psychiatry 2020; 10. doi:10.3389/fpsyt.2019.01007

[9] Julian LJ. Measures of anxiety: State-Trait Anxiety Inventory (STAI), Beck Anxiety Inventory (BAI), and Hospital Anxiety and Depression Scale-Anxiety (HADS-A). Arthritis Care & Research 2011; 63: S467–S472. doi:10.1002/acr.20561

[10] Spielberger CD. State-Trait Anxiety Inventory for Adults. 2012;

[11] Steyer R, Schwenkmezger P, Notz P, et al. Der Mehrdimensionale Befindlichkeitsfragebogen (MDBF) [multidimensional mood questionnaire]. Göttingen, Germany: Hogrefe 1997;

[12] Steyer R, Schwenkmezger P, Notz P, et al. Testtheoretische Analysen des Mehrdimensionalen Befindlichkeitsfragebogen (MDBF). 1994; 40: 320–328

[13] Pruessner JC, Kirschbaum C, Meinlschmid G, et al. Two formulas for computation of the area under the curve represent measures of total hormone concentration versus time-dependent change. Psychoneuroendocrinology 2003; 28: 916–931

[14] Kaye WH, Barbarich NC, Putnam K, et al. Anxiolytic effects of acute tryptophan depletion in anorexia nervosa. International Journal of Eating Disorders 2003; 33: 257–267. doi:10.1002/eat.10135
